# Characteristics of full compensation and its association with total astigmatism: A cross-sectional study

**DOI:** 10.3389/fpubh.2023.1119654

**Published:** 2023-02-06

**Authors:** Ziyun Wu, Yuanyuan Hu, Zihang Xu, Wei Sun, Yirong Wang, Zhen Shao, Yi Liu, Mingkun Yu, Peiran Si, HuanHuan Huo, Xingrong Wang, Hongsheng Bi

**Affiliations:** ^1^Shandong University of Traditional Chinese Medicine, Jinan, China; ^2^Affiliated Eye Hospital of Shandong University of Traditional Chinese Medicine, Jinan, China; ^3^Shandong Academy of Eye Disease Prevention and Therapy, Shandong Provincial Key Laboratory of Integrated Traditional Chinese and Western Medicine for Prevention and Therapy of Ocular Diseases, Shandong Provincial Clinical Research Center of Ophthalmology and Children Visual Impairment Prevention and Control, Shandong Engineering Technology Research Center of Visual Intelligence, Shandong Academy of Health and Myopia Prevention and Control of Children and Adolescents, Jinan, China

**Keywords:** full compensation, total astigmatism, related factors, uncorrected distance visual acuity, cross-sectional study

## Abstract

**Objective:**

To evaluate the characteristics of full compensation and its association with the prevalence of total astigmatism (TA), and to analyze the effects of TA on uncorrected distance visual acuity (UDVA).

**Methods:**

With random cluster sampling based on a school-based cross-sectional design, children aged 4 to 18 years were recruited in September 2020, Shandong Province, China. TA, anterior corneal astigmatism (ACA), and ocular residual astigmatism (ORA) were converted to vectorial components (*J0, J45*), followed by an assessment of the compensatory effect of ACA by ORA. Astigmatism was defined as a cylinder that was better than or equal to 0.75 diopters (D). Logistic regression analysis was used to assess the related factors for children with full compensation, and the generalized linear model was used to assess the influence of TA on UDVA.

**Results:**

Out of 4,494 eligible children, data of 4,145 children (92.3%, 9.23 ± 3.15 years, 50.4% boys) were included in the statistical analysis. The prevalence of TA (27.9%) increased significantly with age (P_trend_ < 0.001). The distribution of full compensation in *J0* and *J45* components were similar (22.1% and 25.6%, respectively), which decreased with age (P_trend_ < 0.001). The closer the refractive status was to emmetropization, the higher the proportion of full compensation and the lower the prevalence of TA were. Shorter axial length (*J0*: Odds Ratio (OR) = 0.76, 95% confidence interval (CI): 0.61 to 0.94, *P* = 0.010), better UDVA (*J0*: OR = 0.37, 95% CI: 0.21 to 0.65, *P* < 0.001; *J45*: OR = 0.34, 95% CI: 0.20 to 0.59, *P* < 0.001), and longer average corneal curvature radius (*J0*: OR = 3.72, 95% CI: 2.18 to 6.34, *P* < 0.001; *J45*: OR = 2.82, 95% CI: 1.67 to 4.76, *P* < 0.001) were associated with full compensation. Higher TA was associated with a worse UDVA (β = 0.03, 95% CI: 0.02 to 0.04, *P* < 0.001).

**Conclusions:**

The prevalence of TA gradually increased with age, and showed a U-shaped distribution with increased refraction. Full compensation was associated with smaller TA and better UDVA. This indicated that considering the compensatory effect of ORA is vital for astigmatism correction in clinical work, which may improve the visual quality.

## Introduction

Astigmatism is a significant and common clinical and public health problem. Uncorrected astigmatism may increase the risk of developing amblyopia and various ocular symptoms (such as glare, monocular diplopia, visual fatigue, and distortion) (1, 2). Total astigmatism (TA) is the result of the combined effect of corneal astigmatism (CA) and ocular residual astigmatism (ORA). CA theoretically consists of anterior corneal astigmatism (ACA) and posterior corneal astigmatism (PCA). However, CA usually refers to ACA. ORA was defined as an astigmatism of posterior corneal surface, plus the crystalline lens astigmatism, and astigmatism caused by aqueous humor.

Previous studies have shown that the cornea was not spherically perfect, and a compensating mechanism between ORA and CA existed (3, 4). In those studies, ACA often exceeded TA, but a balance between internal and corneal optics helped to minimize TA. ORA, however, could not be calculated simply by subtracting ACA from TA unless the astigmatic axis of total and corneal coincide. Instead, Thibos et al. proposed the calculation formula of *J0* and *J45* components (5, 6). Both the magnitude and directional of astigmatism were took into consideration.

The compensatory role of ORA has been already proved to exist. Based on Park and Muftuoglu (3, 7), the ACA of the same magnitude as ORA but in the opposite axial direction was defined as full compensation. However, few studies have assessed the impact on TA by integrating the compensatory effect between ACA and ORA and there is shortage evidence of the related factors about full compensation. In addition, the magnitude of astigmatism might also result in the reduction of uncorrected distance visual acuity (UDVA) and the visual impairment (8). Therefore, our study aimed to analyze the characteristics of compensatory role of ORA, and the associated factors of full compensation in school-aged children, evaluating its influence on the prevalence of TA and UDVA. We hope that these could help understand the general framework of astigmatism occurrence and progression.

## Materials and methods

### Study population

This was a school-based cross-sectional study conducted in Huantai, Shandong, China, in September 2020, which used a multi-stage stratified cluster sampling to recruit children from nine schools (two kindergartens, four primary schools, two middle schools, and one high school). First of all, the local authorities of education provided a list of all schools in Huantai area. Nine schools were then chosen by using convenience sampling. Next, according to the enumeration of grades within the schools, the sampling frame was defined, and ensuring that students aged from 4 to 18 years were included. Finally, classes for each grade level were chosen by simple random sampling. All students in the chosen classes were invited to take part in the research. Children with fundus diseases, cataracts and lens dislocations, or any history of eye surgery, were excluded. Additionally, some individuals with deficient astigmatism data were also excluded from the statistical analysis.

The study was approved by the Ethics committee of the Affiliated Eye Hospital of Shandong University of Traditional Chinese Medicine (HEC-KS-2020016KY). Written consent was obtained from parents and children, and verbal permission was obtained from all participants before the examination.

### Examinations

After supplying questionnaires similar to that used in the previous Refractive Error Study in Children studies to obtain information on parental maternal refractive status, a series of comprehensive ophthalmic examination were carried out by two experienced ophthalmologists. Slit-lamp was the first step to assess anterior and posterior ocular segments, followed by testing UDVA at a distance of 3 meter using the “E” chart (#600722, Good-Lite Co., Elgin, IL, USA). The non-cycloplegic and cycloplegic auto-refractive status of participants were measured by an autorefractor (Nidek ARK-1, CO., LTD, Japan) with consistent parameters (the vertex distance: 12 mm; the measurement step size: 0.25 D). The difference between the maximum and minimum values of spherical and cylindrical degree should be <0.5 D; otherwise, remeasurement was conducted. The cycloplegia was done as follows: one drop of 1% cyclopentolate (Alcon, Fort Worth, TX, USA) was applied to each eye every 5 min for a total of three times. The pupil≥6 mm in diameter was considered as adequate cycloplegia, otherwise, one more drop of cyclopentolate was added and refraction was measured after 10 min. We used IOL-Master 500 (Carl Zeiss Meditec AG, Jena, Germany) to measure Axial length. If the signal-to-noise ratio was <2.1, additional measures were performed until reliable readings were obtained.

Other ophthalmic examination steps have been described in detail in previous researches (9).

### Definition

Astigmatism correction needed in daily life is in the status of natural pupil size (9). Thus, TA and ACA were represented by non-cycloplegic values in the study. Astigmatism is defined as a cylindrical refractive error ≥ 0.75 diopters (D). ACA was obtained by autorefractometry in the range of 3-millimeter corneal diameter and calculated as the difference between the flattest and steepest corneal medians of the anterior corneal surface. The cylindrical axis is equal to the flattest meridian. The sum of the spherical refractive error and half of the cylindrical refractive error was defined as the Spherical Equivalent (SE, expressed as negative values). After cycloplegia, Myopia, pre-myopia, and hyperopia were defined as SE ≤ −0.50 D, −0.50 D < SE ≤ 0.75 D, and SE > 0.75 D, respectively (10). In addition, we classified myopia as mild myopia (−3.00 D < SE ≤ −0.50 D), moderate myopia (−6.00 D < SE ≤ −3.00 D), and high myopia (SE ≤ −6.00 D). We classified hyperopia as mild hyperopia (0.75 D < SE ≤ 2.00 D), moderate hyperopia (2.00 D < SE ≤ 5.00 D), and high hyperopia (SE > 5.00D).

According to Equations 1–2, the cylinder (C), and axis (α) may be converted to power vector (*J0* and *J45* components) (5, 6). C represents negative-cylinder power, and α represents the radians of axis:


(1) $$\begin{array}{l} J\text{0}=\left(-C/2\right)\cos\left(2\alpha \right) \end{array}$$

(2) $$\begin{array}{l} J\text{45}=\left(-C/2\right)\sin\left(2\alpha \right) \end{array}$$

The compensation factor (CF), was calculated as following formulas (Eqs 3–4). ORA*J0*, TA*J0*, and ACA*J0* are the *J0* components of ORA, TA, and ACA, respectively. ORA*J45*, TA*J45*, and ACA*J45* are the *J45* components of ORA, TA, and ACA, respectively.


(3) $$\begin{array}{l} CF0=\left(ACAJ\text{0}-TAJ0\right)/ACAJ\text{0} \end{array}$$

(4) $$\begin{array}{l} CF45=\left(ACAJ\text{45}-TAJ\text{45}\right)/ACAJ\text{45} \end{array}$$

Based on the compensation mechanism of Park and Muftuoglu, CFs were classified as follows: (1) Same axis augmentation: CF< −0.1; (2) No compensation: CF = −0.1 to 0.1; (3) Under compensation: CF = 0.1 to 0.9; (4) Full compensation: CF = 0.9 to 1.1; (5) Over compensation: CF = 1.1 to 2; and (6) Opposite axis augmentation: CF>2 (3, 7).

### Statistical analysis

Statistical analysis was performed by SPSS (SPSS for Windows, version 25.0, Chicago, IL). Only data from the right eyes were chosen for analysis. The Kolmogorov-Smirnov method was used to check the normality of quantitative data. Variables with normal distributions were expressed as mean ± standard deviation (M±SD), unless the median was applied instead. Variables were tested for normality using parametric test, unless non-parametric test was used. Chi-square analysis and P_trend_ values from the Linear-by-Linear Association (LLA) were used to investigate trends in the prevalence of TA and the proportion of full compensation. TA and Full compensation were considered as the dependent variable. Collinearity diagnostics were performed on the independent variables, and those parameters with variance inflation factor (VIF) < 5 were included in the generalized linear model (GLM). Coefficients (β) with 95% confidence intervals (CI) were calculated. All *P*-values were <0.050 were considered statistically significant.

## Result

### Participants

A total of 4,494 children aged 4–18 years were recruited in the cross-sectional study, of whom 349 were excluded (283 with non-cycloplegic refraction, 45 with amblyopia, and 21 with incomplete astigmatism data). The research ultimately included 4,145 (92.3%, 9.23 ± 3.15 years, 50.4% boys) children. As presented in Table 1, boys tended to show a higher magnitude of TA than girls, despite not all age groups reaching a statistically significant level.

**Table 1:** Age and refractive status related changes in TA and ACA.

	**N**	**TA (D)**				**ACA (D)**			
		**Total**	**Boys**	**Girls**	**P** ^*^	**Total**	**Boys**	**Girls**	**P** ^*^
All	4,145	0.25 (0.25, 0.75)	0.50 (0.25, 0.75)	0.25, (0.25, 0.75)	<0.001	1.25 (0.75, 1.50)	1.25 (0.75, 1.50)	1.25 (0.75, 1.50)	0.165
**Age (years)**									
4–7	1,430	0.25 (0.25, 0.50)	0.25 (0.25, 0.50)	0.25 (0.00, 0.50)	0.001	1.00 (0.75, 1.50)	1.00 (0.75, 1.50)	1.00 (0.75, 1.50)	0.629
8–12	1,975	0.50 (0.25, 0.75)	0.50 (0.25, 0.75)	0.25(0.25, 0.75)	<0.001	1.25 (0.75, 1.50)	1.25 (0.75, 1.50)	1.25 (1.00, 1.50)	0.022
13–15	549	0.50 (0.25, 1.00)	0.50 (0.25, 1.25)	0.50 (0.25, 0.75)	<0.001	1.25 (0.75, 1.50)	1.25 (0.75, 1.75)	1.25 (0.75, 1.50)	0.294
16–18	191	0.50 (0.25, 1.00)	0.50 (0.25, 1.25)	0.50 (0.25, 1.00)	0.317	1.25 (1.00, 1.75)	1.13 (0.81, 1.75)	1.25 (1.00, 1.75)	0.956
**Refractive status**									
High hyperopia	10	1.00 (0.50, 2.19)	1.25 (0.44, 2.19)	1.00 (0.63, 2.31)	0.830	1.63 (0.94, 2.81)	2.00 (1.38, 2.94)	0.88 (0.56, 2.50)	0.165
Moderate hyperopia	158	0.50 (0.25, 0.75)	0.50 (0.25, 0.75)	0.50 (0.25, 0.75)	0.297	1.25 (1.00, 1.75)	1.50 (1.00, 2.00)	1.25 (0.75, 1.75)	0.131
Low hyperopia	1,237	0.25 (0.25, 0.50)	0.25(0.25, 0.50)	0.25 (0.06, 0.50)	0.008	1.00 (0.75, 1.50)	1.00 (0.75, 1.50)	1.25 (0.75, 1.50)	0.231
Pre-myopia	1,339	0.25 (0.25, 0.50)	0.25 (0.25, 0.50)	0.25 (0.25, 0.50)	<0.001	1.00 (0.75, 1.50)	1.00 (0.75, 1.50)	1.00 (0.75, 1.50)	0.102
Low myopia	909	0.50 (0.25, 0.75)	0.50 (0.25, 0.75)	0.25 (0.25, 0.75)	<0.001	1.25 (0.75, 1.50)	1.25 (0.75, 1.50)	1.25 (0.75, 1.50)	0.878
Moderate myopia	418	0.75 (0.25, 1.00)	0.75 (0.50, 1.25)	0.75 (0.25, 1.00)	0.002	1.25 (1.00, 1.75)	1.25 (1.00, 1.75)	1.25 (1.00, 1.75)	0.730
High myopia	74	1.25 (0.75, 1.75)	1.50 (1.00, 2.00)	1.00 (0.50, 1.56)	0.008	1.50 (1.00, 2.00)	1.63 (1.25, 2.25)	1.50 (1.00, 2.00)	0.096

TA, Total Astigmatism; ACA, Anterior Corneal Astigmatism; D, Diopter. ^*^, Mann Whitney Wilcoxon Test.

**Figure 1:**
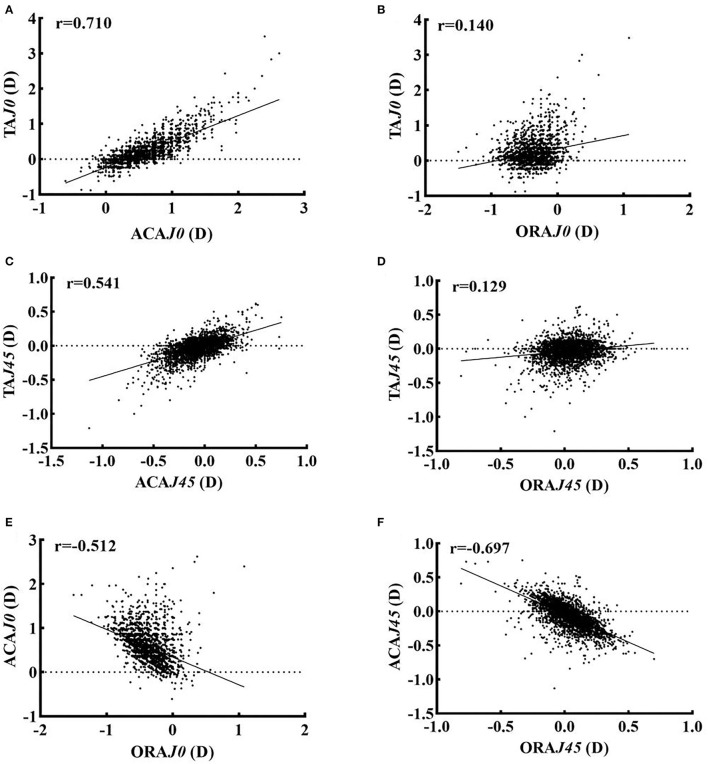
Spearman correlation analysis were used to analyze the correlation of TA, ACA, and ORA in *J0*, and *J45* components. **(A)** ACA *J0* vs. TA *J0*. **(B)** ORA *J0* vs. TA *J0*. **(C)** ACA *J45* vs. TA *J45*. **(D)** ORA *J45* vs. TA *J45*. **(E)** ACA *J0* vs. ORA *J0*. **(F)**. ACA *J45* vs. ORA *J45*. *J0* and *J45* components represent the orthogonal (power of Jackson cross cylinder at 90° and 180°) and oblique (power of the Jackson cross cylinder at 45° and 135°), respectively. TA, Total Astigmatism; ACA, Anterior Corneal Astigmatism; ORA, Ocular residual astigmatism; D, diopter.

The prevalence was 27.9% (1157/4145) for TA and 86.7% (3594/4145) for ACA, respectively (χ^2^ = 139.50, *P* < 0.001). Spearman correlation analysis in Figure 1 showed positive correlations between TA*J0* and ACA*J0* (*r* = 0.710, *P* < 0.001), TA*J0* and ORA*J0* (*r* = 0.140, *P* < 0.001), TA*J45* and ACA*J45* (*r* = 0.541, *P* < 0.001), and TA*J45* and ORA *J45* (*r* = 0.129, *P* < 0.001). However, there was a negative correlation between ACA and ORA for both *J0* (*r* = −0.512, *P* < 0.001) and *J45* (*r* = −0.697, *P* < 0.001) components, suggesting the existence of a compensatory mechanism for ACA by ORA.

### Compensation factor

**Figure 2:**
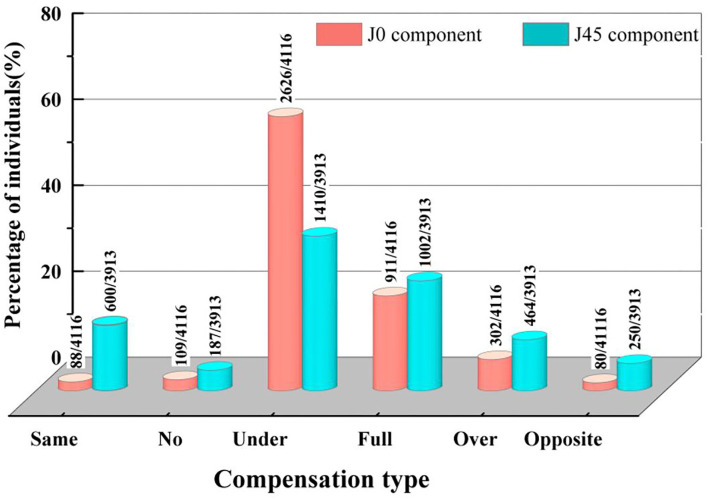
Distribution of compensation factor types in *J0* and *J45* components. *J0* and *J45* components represent the orthogonal (power of Jackson cross cylinder at 90° and 180°) and oblique (power of the Jackson cross cylinder at 45° and 135°), respectively. The percentage of compensation types in *J0, J45* components are shown on the y-axis. The groups of compensation types are shown on the x-axis. (1) Same: same axis augmentation; (2) No: no compensation; (3) Under; under compensation; (4) Full: full compensation; (5) Over: over compensation; (6) Opposite: opposite axis augmentation.

In *n* = 29 for *J0* and *n* = 233 for *J45* of ACA, CF was not determined as the denominator was zero. CF percentages of all children were summarized in Figure 2. Most of the compensation types were under compensation and full compensation (*J0*: 85.9%; *J45*: 61.6%), indicating TA fell below ACA, but the astigmatism axis remained the same. The percentages of full compensation in the *J0* and *J45* components (22.1 and 25.6%, respectively) were similar.

### Correlations between the proportion of full compensation and the prevalence of TA with age and refractive status

**Figure 3:**
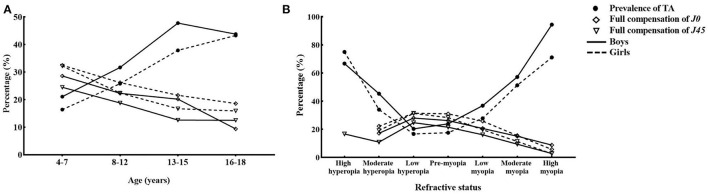
Age **(A)** and refractive status **(B)** related changes in full compensation and total astigmatism. *J0* and *J45* components represented the orthogonal (power of Jackson cross cylinder at 90° and 180°) and oblique (power of the Jackson cross cylinder at 45° and 135°), respectively. The percentage of TA and full compensation in *J0, J45* components are shown on the y-axis. The groups of age and refractive status are shown on the x-axis. After cycloplegia, pre-myopia was defined as−0.50 D < SE ≤ 0.75 D. We classified myopia as mild myopia (−3.00 D < SE ≤ −0.5 D), moderate myopia (−6.00 D) < SE ≤ 3.00 D, and high myopia (SE ≤ −6.00 D) after cycloplegia. We classified hyperopia as mild myopia (−0.75 D < SE ≤ 2.00 D), moderate myopia (2.00 D < SE ≤ 5.00 D), and high hyperopia (SE > 5.00 D). TA, Total Astigmatism; D, Diopter.

Figure 3 suggests that boys tend to show a higher prevalence of TA than girls. For *J0* and *J45* components, Figure 3A presents that the proportion of full compensation decreases significantly with age (P_trend_ < 0.001), and the prevalence of TA increased significantly with age (P_trend_ < 0.001). The prevalence of TA in children varied with refractive status in a U-shaped distribution (Figure 3B). The closer the refractive status was to emmetropization, the higher the proportion of full compensation and the lower the prevalence of TA were.

### Related factors of full compensation

For *J0* and *J45* components, comparisons for related factors about full compensation were shown in Table 2. Children with full compensation were more likely to be younger and associated with larger cycloplegic SE, shorter axial length, longer average anterior corneal curvature radius, better UDVA, and smaller TA and ACA (*P* < 0.001).

**Table 2:** Comparison of eye parameters for whether full compensation.

	***J0*** **component (Whether full compensation)**			***J45*** **component (Whether full compensation)**		
	**No**	**Yes**	***P*** **-value**	**No**	**Yes**	***P*** **-value**
**Gender**						
Boys	1,659 (80.1%)	411 (19.9%)	<0.001	1,483 (76.0%)	468 (24.0%)	0.021
Girls	1,546 (75.6%)	500 (24.4%)		1,428 (72.8%)	534 (27.2%)	
**Age (years)**	9.00 (7.00, 12.00)	8.00 (6.00, 10.00)	<0.001	9.00 (7.00, 12.00)	8.00 (7.00, 10.00)	<0.001
Cycloplegic SE (D)	0.25 (−1.50, 1.00)	0.62 (−3.78, 1.13)	<0.001	0.25 (−1.38, 1.00)	0.63 (−0.50, 1.13)	<0.001
Axial length (mm)	23.38 (22.66, 24.34)	23.13 (22.55, 23.80)	<0.001	23.36 (22.63, 24.29)	23.24 (22.59, 23.95)	<0.001
Average anterior corneal curvature radius (mm)	7.78 (7.63, 7.96)	7.82 (7.64, 7.99)	<0.001	7.78 (7.61, 7.96)	7.83 (7.67, 7.99)	<0.001
Intraocular pressure (mmHg)	16.00 (15.00, 18.00)	16.00 (14.00, 18.00)	0.339	16.00 (15.00, 18.00)	16.00 (14.00, 18.00)	0.193
UDVA (log MAR)	0.00 (0.00, 0.40)	0.00 (0.00, 0.10)	<0.001	0.00 (0.00, 0.40)	0.00 (0.00, 0.10)	<0.001
Total astigmatism (D)	0.50 (0.25, 0.75)	0.00 (0.00, 0.00)	<0.001	0.50 (0.25, 0.75)	0.00 (0.00, 0.25)	<0.001
Anterior corneal astigmatism (D)	1.25 (1.00, 1.75)	1.00 (0.75, 1.25)	<0.001	1.25 (1.00, 1.75)	1.00 (0.75, 1.25)	<0.001

D, Diopter; mm, Millimeter; mmHg, Millimeter of mercury; UDVA, Uncorrected Distance Visual Acuity.

As shown in Table 3, multiple logistic regression was used to assess the related factors of full compensation (the univariate logistic regression shown in Supplementary Table 1). Better UDVA (*J0*: OR = 0.37, 95% CI: 0.21 to 0.65; *P* = 0.001; *J45*: OR = 0.34, 95% CI: 0.20 to 0.59, *P* < 0.001), shorter axial length (*J0*: OR = 0.76, 95% CI: 0.61 to 0.94, *P* < 0.01), and longer average anterior corneal radius (*J0*: OR = 3.72, 95% CI: 2.18 to 6.34, *P* < 0.001; *J45*: OR = 2.82, 95% CI: 1.67 to 4.76, *P* < 0.001) were associated with full compensation.

**Table 3:** Multivariate Logistic regression analysis assessing related factors for children with full compensation.

	***J0*** **component**		***J45*** **component**	
	**OR (95%CI)**	***P*** **-value**	**OR (95%CI)**	***P*** **-value**
Gender _ Boys	0.69 (0.58,0.82)	<0.001	0.72 (0.61,0.85)	<0.001
Age (years)	0.94 (0.91,0.98)	0.003	0.96 (0.93,1.00)	0.047
Cycloplegic SE (D)	0.88 (0.79,0.99)	0.039	0.93 (0.83,1.04)	0.198
Axial length (mm)	0.76 (0.61,0.94)	0.010	0.90 (0.73,1.11)	0.322
Average anterior corneal curvature radius (mm)	3.72 (2.18,6.34)	<0.001	2.82 (1.67,4.76)	<0.001
UDVA (Log MAR)	0.37 (0.21,0.65)	<0.001	0.34 (0.20,0.59)	<0.001

D, Diopter; mm, millimeter; UDVA, uncorrected distance visual acuity.

### Correlations between full compensation, TA, and UDVA

As shown in Table 4, TA was determined as the dependent variable in model 1, and UDVA was determined as the dependent variable in models 2 and 3. The univariate GLM could be seen in Supplementary Table 2. After adjusting for age, gender, cycloplegic SE, and other factors. Multivariate GLM showed that children with full compensation may contribute to a smaller TA in model 1 (*J0*: β = −0.42, 95% CI: −0.47 to −0.37, *P* < 0.001; *J45*: β = −0.18, 95% CI: −0.23 to −0.14, *P* < 0.001) and better UDVA in model 2 (*J45*: β = −0.02, 95% CI: −0.04 to −0.00, *P* = 0.015). Moreover, larger TA was associated with worse UDVA in model 3 (β = 0.03, 95% CI: 0.02 to 0.04, *P* < 0.001).

**Table 4:** Effect of full compensation on total astigmatism and uncorrected distance visual acuity based on multivariate Generalized Linear Model.

	**Model 1**		**Model 2**		**Model 3**	
	β **(95%CI)**	*P* ^*^ **-value**	β **(95%CI)**	*P* ^*^ **-value**	β **(95%CI)**	*P* ^*^ **-value**
Full compensation of *J0* component	−0.42 (−0.47, −0.37)	<0.001	−0.01 (−0.03, 0.01)	0.039	–	
Full compensation of *J45* component	−0.18 (−0.23, −0.14)	<0.001	−0.02 (−0.04, −0.00)	0.015	–	
Total astigmatism (D)	–		–		0.03 (0.02, 0.04)	<0.001
Gender _ Boys	0.08 (0.05, 0.11)	<0.001	−0.03 (−0.04, −0.02)	<0.001	−0.03 (−0.04, −0.02)	<0.001
Age (years)	0.00 (0.00, 0.01)	0.268	0.01 (0.01, 0.01)	<0.001	0.01 (0.01, 0.01)	<0.001
Cycloplegic SE (D)	−0.05 (−0.06, −0.04)	<0.001	−0.01 (−0.01, −0.09)	<0.001	−0.09 (−0.10, −0.09)	<0.001

Model 1: Total astigmatism was the dependent variable; Model 2 and Model 3: Uncorrected distance visual acuity (LogMAR acuity) was the dependent variable.

P^*^-Values were calculated with a multivariate Generalized Linear Model adjusted for age, gender, cycloplegic spherical equivalence, maternal and parental refractive status.

D, Diopter; SE, spherical equivalence.

## Discussion

Using cross-sectional data in Shandong, China, we firstly explored the related factors of full compensation and found the effects of astigmatism on visual acuity. The results showed that children with full compensation had smaller TA. Yet, higher TA was associated with worse UDVA in children after adjusting for age, gender, spherical powers, and parental refractive status. Our study also provided new information on astigmatism distribution in children aged 4–18: the proportions of full compensation in the *J0* and *J45* components were 22.1 and 25.6%, and the prevalence of TA was 27.9%, respectively. However, Park et al. investigated 178 adults (aged 19–46 years) and examined the compensation of ORA. They found that for the *J0* component, 4% was full compensation, and for the *J45* component, 12% was full compensation (3). Their percentages were lower than those in our research in both the *J0* and *J45* components. This difference might be attributable to the age effect. We found that the efficiency of full compensation decreased with age in children aged 4–18 years. The phenomenon may be related to ocular development and myopic progression. The cornea is not perfectly spherical. ACA, PCA, the crystalline lens, the asymmetry of each refractive error component of the eye, the tear film conditions, and intrinsic variation of the refractive index, etc., are the complex factors that contribute to astigmatism (11). A disruption of any factor could affect the compensation mechanism.

CA theoretically is the combination of ACA and PCA. However, because of the difficulties in measuring PCA and the relatively small influence on TA, CA generally only refers to ACA. PCA was considered ORA in most cases. In addition, other components such as aqueous humor, crystalline lens, and vitreous body contribute to ORA (12), which may help neutralize or offset a portion of the ACA to diminish TA or superimpose with ACA to increase TA. Identifying the compensatory mechanism of ORA can help us understand the distribution of astigmatism and screen children who need early intervention. It also assists clinicians in designing post-operative RA, reducing unnecessary ORA after corneal remodeling. For example, when ORA exhibits an offsetting effect on ACA, leaving the ACA of the same magnitude as ORA but in the opposite axial direction should be considered in kerato-refractive laser surgery, which may improve post-operative vision.

Children with full compensation had better UDVA in the *J45* component (Shown in Table 4), and the larger TA was associated with worse UDVA (model 3, shown in Table 4). Similar findings also suggested a positive association between visual acuity and astigmatism (LogMAR acuity = 0.068 + 0.055 astigmatism) (13). Moreover, a case-control study revealed that low-level ORA induced by IOL implantation in patients also reduced their visual acuity (14). The reason is that astigmatism could prevent the human eye from focusing complex visual information on the retina, leading to blurred vision. Continuing to find or eliminate the reasons for ORA remains a goal and a challenge for clinical physicians in precision medicine. The balance between the optics in the eye and the cornea helps minimize ORA. ORA is often unpredictable and affects visual quality. Even excellent ortho-K lens fitting may lead to irregular ORA. This can even result in severe visual discomforts such as double vision, glare, metamorphopsia, and decreased visual quality (15). The accuracy of refractive data measurement and calculation needs to be continuously improved. Therefore, highlighting the need for astigmatism correction, based on the compensation mechanism, is important in clinical work and research.

Our study found that the magnitude and prevalence of TA showed a U-shaped distribution with refractive status. A similar study showed that children with myopia and hyperopia tended to develop astigmatism (16). In other words, children with refractive errors are more likely to have astigmatism than those without refractive errors. This phenomenon may be related to many factors. When children are young, they have a higher hyperopia reserve and present higher astigmatism. When children grow up, myopia progresses. The increase in axial length (a combined parameter about the chamber depth, and lens thickness, with vitreous chamber depth) may lead to changes in ORA (e.g., lens tilt). This could disrupt the attenuation of TA and thus cause an increase in TA. Correcting astigmatism may be an effective way to prevent and control refractive errors in children.

Astigmatism is associated with a significant social and financial burden all over the world. Though the precise cost of astigmatism treatment was unavailable, it was reported that the direct cost of myopia for each subject was $221.68 for Singapore school children (17). To reduce the effect of astigmatism, the accuracy of refractive data correction needs to be continuously improved in clinical work. Therefore, optometrists or ophthalmologists pay attention to the balance between the eye's optics and cornea to minimize TA.

The strengths of this study were that we use Fourier analysis to transform refractive clinical data into vector notation, which considered both the magnitude and axis of astigmatism. Then, we identified the risk factors for children with full compensation, and identified the relationship between TA and UDVA. Unfortunately, we did not measure the contribution of the internal structure of the ORA (such as the anterior chamber depth, and thickness of the crystal, or lens tilt) to the TA. We were also unable to assess the contribution of PCA on TA due to the difficulty of measuring PCA. At last, the compensatory effect of ORA and ACA on visual acuity was also significantly correlated with other factors (such as higher-order aberrations, pupil size, accommodation, and cortical modulation of astigmatism). These factors could not be further investigated due to the limitations of epidemiological investigations. Long-term longitudinal data are needed to provide more convincing evidence due to the limitations of cross-sectional studies.

In conclusion, we found the related risk factors of full compensation. Children with full compensation had better UDVA and smaller TA. In order to reduce the impact of TA, an emphasis on astigmatism vector analysis or compensatory efficiency is required, especially in corneal altering procedures (such as refractive surgeries and orthokeratology lens fitting) or IOL lens implantation.

## Data availability statement

The raw data supporting the conclusions of this article will be made available by the authors, without undue reservation.

## Ethics statement

The studies involving human participants were reviewed and approved by the Ethics Committee of the Affiliated Eye Hospital of Shandong University of Traditional Chinese Medicine (HEC-KS-2020016KY). Written informed consent to participate in this study was provided by the participants' legal guardian/next of kin.

## Author contributions

HB and XW had full access to all the data in the study and takes responsibility for the integrity of the data and accuracy of the data analysis and conceptualized this study. HB supervised the study. ZW, YH, ZX, WS, YW, ZS, YL, MY, PS, and HH collected data for this study. ZW performed the statistical analyses. ZW, YH, and ZX drafted the manuscript. All authors contributed to the critical revision of the manuscript and approved the final manuscript.
