# Supplementary material for: Characteristics of full compensation and its association with total astigmatism: A cross-sectional study
Source: Front Public Health. 2023 Feb 6;11:1119654. doi: 10.3389/fpubh.2023.1119654 (PMC9939449; doi:10.3389/fpubh.2023.1119654)
Supplement: Supplementary file 1 [file Table_1.DOCX]

**Supplement table 1** Univariate Logistic regression analysis assessing related factors for children with full compensation

|  | ***J0* component** | |  | ***J45* component** | |
| --- | --- | --- | --- | --- | --- |
|  | **OR (95%CI)** | **P value** |  | **OR (95%CI)** | **P value** |
| **Gender _ Boys** | 0.77（0.66，0.89） | <0.001 |  | 0.84(0.73, 0.97) | 0.021 |
| **Age(years)** | 0.90（0.88，0.92） | <0.001 |  | 0.93（0.91，0.96） | <0.001 |
| **Cycloplegic SE (D)** | 1.18（0.14，1.23） | <0.001 |  | 1.13（0.09，0.17） | <0.001 |
| **Axial length (mm)** | 0.80（0.76，0.86） | <0.001 |  | 0.89（0.83，0.94） | <0.001 |
| **Average anterior corneal curvature radius(mm)** | 1.62（1.20，2.18） | 0.001 |  | 1.88（0.40，0.51） | <0.001 |
| **UDVA (Log MAR)** | 0.22（0.16，0.31） | <0.001 |  | 0.31（0.22，0.42） | <0.001 |

Abbreviations: D=Diopter; mm= millimeter; UDVA= uncorrected distance visual acuity.
