# Supplementary material for: Characteristics of full compensation and its association with total astigmatism: A cross-sectional study
Source: Front Public Health. 2023 Feb 6;11:1119654. doi: 10.3389/fpubh.2023.1119654 (PMC9939449; doi:10.3389/fpubh.2023.1119654)
Supplement: Supplementary file 2 [file Table_2.DOCX]

**Supplement table 2** Effect of full compensation on total astigmatism and uncorrected distance visual acuity based on univariate Generalized Linear Model

|  | **Model 1** | |  | **Model 2** | |  | **Model 3** | |
| --- | --- | --- | --- | --- | --- | --- | --- | --- |
|  | **β (95%CI)** | **P^*^value** |  | **β (95%CI)** | **P^*^value** |  | **β (95%CI)** | **P^*^ value** |
| **Full compensation of *J0* component** | -0.60(-0.63,-0.56) | ＜0.001 |  | -0.09(-0.11,-0.07) | ＜0.001 |  | - |  |
| **Full compensation of *J45* component** | -0.50(-0.53,-0.46) | ＜0.001 |  | -0.08(-0.10,-0.06) | ＜0.001 |  | - |  |
| **Total astigmatism (D)** | - | ＜0.001 |  | - | ＜0.001 |  | 0.13(0.11,0.14) | ＜0.001 |
| **Gender _ Boys** | 0.10(0.06,0.10) | ＜0.001 |  | -0.04(-0.06,-0.03) | ＜0.001 |  | -0.04(-0.06,-0.03) | ＜0.001 |
| **Age (years)** | 0.04(0.03,0.04) | ＜0.001 |  | 0.05(0.05,0.05) | ＜0.001 |  | 0.05(0.05,0.05) | ＜0.001 |
| **Cycloplegic SE (D)** | -0.07(-0.07,-0.07) | ＜0.001 |  | -0.10(-0.11,-0.10) | ＜0.001 |  | -0.10(-0.11,-0.10) | ＜0.001 |
| **Paternal refractive status** |  |  |  |  |  |  |  |  |
| Emmetropia | Ref |  |  | Ref |  |  | Ref |  |
| Mild or moderate myopia | -0.02(-0.06,-0.02) | 0.247 |  | -0.01(-0.03,0.01) | 0.354 |  | -0.01(-0.03,0.01) | 0.354 |
| High myopia | 0.03(-0.04,0.03) | 0.411 |  | 0.07(0.03,0.10) | ＜0.001 |  | 0.07(0.03,0.10) | ＜0.001 |
| Hyperopia | 0.11(-0.05,0.11) | 0.178 |  | 0.08(0.00,0.16) | 0.04 |  | 0.08(0.00,0.16) | 0.04 |
| **Maternal refractive status** |  |  |  |  |  |  |  |  |
| Emmetropia | Ref |  |  | Ref |  |  | Ref |  |
| Mild or moderate myopia | -0.02(-0.06,-0.02) | 0.298 |  | -0.01(-0.03,0.01) | 0.367 |  | -0.01(-0.03,0.01) | 0.367 |
| High myopia | 0.08(0.00,0.08) | 0.044 |  | 0.10(0.06,0.13) | ＜0.001 |  | 0.10(0.06,0.13) | ＜0.001 |
| Hyperopia | -0.04(-0.22,-0.04) | 0.648 |  | 0.11(0.02,0.20) | 0.019 |  | 0.11(0.02,0.20) | 0.019 |

Model1: Total astigmatism was the dependent variable; Model2 and Model3: Uncorrected distance visual acuity (LogMAR acuity) was the dependent variable;

P* Values were calculated with a univariate Generalized Linear Model.

Abbreviations: D=Diopter; SE= spherical equivalence.
